# Whole Blood Interferon γ Release Is a More Sensitive Marker of Prior Exposure to *Coxiella burnetii* Than Are Antibody Responses

**DOI:** 10.3389/fimmu.2021.701811

**Published:** 2021-07-28

**Authors:** Anja Scholzen, Margot de Vries, Hans-Peter Duerr, Hendrik-Jan Roest, Ann E. Sluder, Mark C. Poznansky, Milou L. C. E. Kouwijzer, Anja Garritsen

**Affiliations:** ^1^Innatoss Laboratories B. V., Oss, Netherlands; ^2^Numerus GmbH, Tübingen, Germany; ^3^Department of Bacteriology and Epidemiology, Wageningen Bioveterinary Research, Lelystad, Netherlands; ^4^Vaccine and Immunotherapy Center, Massachusetts General Hospital, Boston, MA, United States

**Keywords:** Coxiella *burnetii*, Q fever, human, T-cell, interferon gamma, exposure, biomarker, diagnostic test

## Abstract

For the zoonotic disease Q fever, serological analysis plays a dominant role in the diagnosis of *Coxiella burnetii* infection and in pre-screening for past exposure prior to vaccination. A number of studies suggest that assessment of *C. burnetii*-specific T-cell IFNγ responses may be a more sensitive tool to assess past exposure. In this study, we assessed the performance of a whole blood *C. burnetii* IFNγ release assay in comparison to serological detection in an area of high Q fever incidence in 2014, up to seven years after initial exposure during the Dutch Q fever outbreak 2007-2010. In a cohort of >1500 individuals from the Dutch outbreak village of Herpen, approximately 60% had mounted IFNγ responses to *C. burnetii*. This proportion was independent of the *Coxiella* strain used for stimulation and much higher than the proportion of individuals scored sero-positive using the serological gold standard immunofluorescence assay. Moreover, *C. burnetii*-specific IFNγ responses were found to be more durable than antibody responses in two sub-groups of individuals known to have sero-converted as of 2007 or previously reported to the municipality as notified Q fever cases. A novel ready-to-use version of the IFNγ release assay assessed in a subgroup of pre-exposed individuals in 2021 (10-14 years post exposure) proved again to be more sensitive than serology in detecting past exposure. These data demonstrate that *C. burnetii*-induced IFNγ release is indeed a more sensitive and durable marker of exposure to *C. burnetii* than are serological responses. In combination with a simplified assay version suitable for implementation in routine diagnostic settings, this makes the assessment of IFNγ responses a valuable tool for exposure screening to obtain epidemiological data, and to identify previously exposed individuals in pre-vaccination screens.

## Introduction

The zoonotic disease Q fever is caused by the environmentally highly stable small Gram-negative coccobacillus *Coxiella burnetii* (1). Outbreaks usually occur in occupational settings such as the livestock industry and deployed military personnel (1), but also affect the general population, exemplified by the largest reported Q fever outbreak, which occurred in the Netherlands from 2007-2010 with an estimated 40,000 infections (2). Approximately 50-60% of infected individuals remain asymptomatic and acute infection is readily treatable using antibiotics or self-limiting. However, years after infection, 1-5% of infected individuals with specific risk factors progress to chronic Q fever (1, 3). Based on a meta-analysis of cohorts mostly from Australia, North America and Europe, approximately 20% of those with symptomatic disease suffer from a debilitating fatigue (post Q fever chronic fatigue syndrome) for more than 12 months after acute infection, with a major long-term impact on quality of life (4) and considerable economic consequences (5). Given these risks and associated economical costs, in Australia high risk individuals are strongly advised to be vaccinated with the whole-cell formalin-inactivated vaccine Q-VAX, the only vaccine licensed for human use to date (6, 7). This vaccine is highly effective in pre-exposure prophylaxis, but requires screening for prior exposure to *C. burnetii* to limit reactogenicity and is not registered outside Australia (6–9).

Direct detection of *C. burnetii* by real-time PCR is used to confirm infection within two weeks after acute infection when individuals are still sero-negative (10), and in conjunction with clinical data and imaging of sites of infection in the diagnosis of persistent infection/chronic Q fever (1, 11). Serological analysis of anti-*Coxiella* antibodies, however, plays the dominant role in the diagnosis of *C. burnetii* infection as well as for pre-screening for past exposure (1). Of note, the sensitivity of the various serological tests used interchangeably can vary across different laboratories within and across countries: The standard immunofluorescence assay (IFA) is more reliably positive one year after acute infection or in chronic Q fever than are the ELISA and complement fixation test (CFT) (12, 13), but can suffer greatly from differences in interpretation between operators (14, 15). Furthermore, the absence of detectable anti-*Coxiella* antibodies even by IFA does not exclude past infection or exposure: Following acute infection, the half-life of IgG phase 2 antibodies is extrapolated to be 318 days (16), and approximately 20% of patients become seronegative after 6 years (17). In Australia, vaccination pre-screening relies not just on serology but also implements a skin test equivalent to the tuberculin skin test to evaluate cellular responses to *C. burnetii* (9, 18). Correspondence between the serological and skin tests is poor (19, 20) and a significant proportion of vaccinated individuals who are negative in these pre-screens still experience adverse reactions, particularly younger adults < 50 years of age and females (19, 21): local adverse reactions occur in 80-98% (20-30% grade 3-4/severe to extreme) and systemic adverse events in 50-60% of vaccinees (ca. 5% grade 3-4/severe to extreme) (19, 21). A whole blood stimulation assay using heat-killed whole cell *C. burnetii* (22) detected cellular IFNγ responses in a considerable proportion of elderly Dutch individuals with cardiovascular risk factors who passed the pre-vaccination screening with both negative serology (by IFA) and skin test results (23). In this cohort, there was also a trend for more common local adverse reactions to the formalin-inactivated whole cell vaccine in those with high pre-vaccination IFNγ responses (19).

These data indicate that assessment of cellular responses to *C. burnetii* by IFNγ release assay (IGRA) provides a valuable additional tool to evaluate (past) infection, one which may be more sensitive than either serological detection or the invasive skin-test. In the present study we therefore determined the prevalence and durability of cellular IFNγ responses to *C. burnetii* using a *C. burnetii* IGRA in comparison to serological detection in two cohorts of individuals, one from an area of past wide-spread exposure (>1500 individuals) and one from an area of lower prevalence (n=109) in the Netherlands, up to seven years after initial exposure and four years after the Dutch Q fever outbreak was resolved. To facilitate implementation in a routine diagnostic setting, we further developed a simplified, ready-to-use version of the *C. burnetii* IGRA and assessed its performance in 2021, ten to fourteen years after initial exposure, in a subgroup of n=95 individuals with known past exposure and known IGRA and/or IFA data from 2014.

## Materials and Methods

### Study Population

A large cross-sectional population study was performed in collaboration with the municipal health service (Gemeentelijke GezondheidsDienst, GGD) “Hart voor Brabant” and the Jeroen Bosch Hospital (‘s-Hertogenbosch, Netherlands) as part of the Q Herpen II project in which 70% of the adult population of the village of Herpen were screened for exposure to the *C. burnetii* (24, 25). This village in the Dutch province of North Brabant experienced a high incidence of *C. burnetii* infection during the 2007-2010 Q fever outbreak (26). The medical ethics review committee of the University Medical Center Utrecht approved the study (Q Herpen II, NL45224.041.13, protocol 13-367/D). In January 2014, all n=2161 adult inhabitants were invited to participate. Spread over 6 days in February and March 2014, n=1515 individuals provided their written informed consent to participate in this study and filled in a questionnaire providing self-reported information regarding demographics, smoking history, risk factors associated with chronic Q fever, history of Q fever infection (notified and self-reported) and vaccination. Informed consent forms and questionnaires were checked for missing information and errors by medical staff and the participant, followed by a venipuncture for assessment of Q fever serology and cellular responses.

Additional blood samples were collected in November 2014 from a group of n=109 individuals from the low incidence area of Enschede in the Dutch Province of Overijssel, which experienced a much lower incidence of Q fever during the outbreak than Noord-Brabant. Visitors to a blood-drawing facility were asked to participate in the study as part of a planned visit. A group of n=16 known former Q fever patients were invited to a parallel track, in order to be able to verify an novel immunoblot procedure used to establish formation of antibodies. This study was approved by the medical ethics review committee Brabant (NL50415.028.14) and all subjects provided written informed consent.

In January 2021, n=105 individuals with known prior *Coxiella* exposure status from earlier studies were enrolled for reassessment of their cellular responses using the new ready-to-use format of the Q-detect™ IGRA. Amongst those, n=95 individuals were available for a blood draw, including n=84 individuals from the initial Q Herpen II study. This study was approved by the medical ethics review committee Brabant (NL74801.028.20) and all participants provided written informed consent.

Samples from n=98 de-identified blood bank donors from the Amsterdam/Rotterdam area were obtained in November 2020 from the Sanquin Bloodbank under agreement number NVT0417.01 for technical validation and determination of cut-off criteria of the ready-to-use IGRA format.

### *C. burnetii* Antigen Preparations

Three different antigens were used for whole blood stimulations, the reference strain *C. burnetii* Nine Mile RSA 493 originally isolated from a tick (27) as well as two *C. burnetii* strains isolated from the placenta of goats infected during the Dutch outbreak, *C. burnetii* 2009-02629 (Cb2629; the proprietary Q-detect™ antigen) and X09003262 (Cb03262) (28, 29). All three strains carry the phase I LPS variant and the QpH1 plasmid and show the same CbNL01 genotype determined by multi-locus variable-number tandem-repeat analyses (MLVA) (28, 29). For the reference strain Nine Mile RSA 493, kindly provided by D. Frangoulidis (Bundeswehr Institute of Microbiology, Munich, Germany), the same heat-killed batch as in the original study (22) was used. This batch of *C. burnetii* Nine Mile was grown on monolayers of Buffalo Green Monkey (BGM) cells, a time-consuming culture method that is difficult to scale up and reproduce, and prone to contamination with BGM cell debris, all of which are undesirable when producing antigen batches for a diagnostic assay. Therefore, separate batches of heat-killed *C. burnetii* whole cell antigen from strains Cb02629 (lot 14VRIM014) and Cb03262 were prepared from a master cell bank using a cell-free culture method at the Central Veterinary Institute, Wageningen Bioveterinay Research, Lelystad, The Netherlands (30–32). Both batches were quality controlled including determination of the protein concentration using Western Blot, TNFα release by THP-1 cells as a measure for functional TLR stimulation and dose-response titrations assessing IFNγ release in known Q fever exposed individuals.

### Serological Responses to *C. burnetii*

IgG and IgM antibody titers for phase I and phase II *C. burnetii* were determined by immunofluorescence assay (Focus Diagnostics) at the Jeroen Bosch Hospital, ‘s-Hertogenbosch, the Netherlands. In the Q Herpen II study, IgG phase I or II titer of ≥1:64 was interpreted as IFA-positive, and phase II endpoint titers were only determined for those samples with a positive IgG phase I result, as reported previously (25). For the 2021 study, IgG phase I and II titers were determined for all samples up to a dilution of 1:256 to also allow analysis according to the manufacturers’ instructions.

In the Enschede cohort, serological responses were additionally assessed by an in-house immunoblot using Cb2629 as the target antigen. Briefly, Cb2629 was separated using 10% NUPAGE Bis-Tris gels (Thermo Fisher) and blotted onto a PVDF membrane (Thermo Fisher). Immunoblot strips were cut, blocked with 5% skim milk in Dulbecco’s PBS/0.1% Tween 20 and incubated with 50-fold diluted serum samples. Strips were developed using alkaline phosphatase-conjugated goat anti-Human IgG and BCIP/NBT chromogenic substrate (Thermo Fisher). Positive bands were evaluated visually. This immunoblot identified 9/9 IFA positive serum samples from donors with known prior Q fever tested in parallel, and detected *C. burnetii*-specific IgG at a 5-fold lower serum dilution than IFA (data not shown).

### Whole Blood IFNγ Release Assay (Q-Detect™)

The Q-detect™ IGRA was adapted from the protocol used in a previous study (22), standardized and optimized for high throughput (see **Supporting Information Part 1**). Undiluted whole lithium-heparin anti-coagulated blood was stimulated within 8 hours from blood collection with heat-killed whole cell *C. burnetii* antigen in 96-well polypropylene plates (Greiner BioOne) by adding 180 µl blood to a solution of 20 µl containing *C. burnetii* antigen pre-diluted in phenol red-free RPMI supplemented with Glutamax (2 mM), Gentamycin (5 µg/ml) and sodium pyruvate (1 mM, all Thermo Fisher). A 1.5% (v/v, final concentration) solution of PHA-M (Thermo Fisher, Cat. No. 10576015), was added to separate wells as a positive control. Medium only was added to the negative control wells. All stimulations were performed in duplicate. After 21-23 hours incubation at 37°C, whole blood cultures were re-suspended and IFNγ concentrations were assessed in whole blood by ELISA, using the IFNγ Pelipair protocol (Sanquin) with minor modifications. Negative control and *C. burnetii*-stimulated samples were assayed in 2-fold dilution and positive control samples were assayed in 5-fold dilution. Concentrations were calculated using a standard curve obtained by four parameter logistic curve fitting. Negative control responses that were too low to be calculated were assigned a concentration of 0.6 pg/mL, which is the limit of detection of the ELISA. This detection limit was based on 180 calibration curves assessed during the present study. Concentrations above the range of the standard curve were assigned 105% of the concentration of the highest standard (500 pg/mL) multiplied by the dilution factor. The upper detection limit of IGRA under these conditions is 1050 pg/ml for *C. burnetii* and 2625 pg/mL for the positive control. Duplicates were averaged following log-transformation (i.e. using the geometric mean). A subject was scored positive by IGRA if negative and positive controls met the quality cut-offs (**Supporting Information Part 1, Supporting Figure S1**), the *C. burnetii*-induced IFNγ production was ≥16 pg/ml above background and the *Coxiella* relative ratio (CoxRR) of the logarithmic value of background-subtracted *C. burnetii* and PHA responses [(log(C. burnetii)-log[neg control])/(log(PHA)-log(neg control))] was ≥0.4 (**Supporting Figure S2**).

### Ready-to Use Format of the Whole Blood IFNγ Release Assay (Q-detect™ 2.0)

The Q-detect™ IGRA was adapted to a new ready-to-use format combining microtubes pre-coated with heat-killed whole cell *C. burnetii* Cb2629 antigen or Staphylococcus enterotoxin B (Sigma, Cat. No. S4881) as a positive control, both deposited in a sucrose matrix, combined with a fully validated in-house ready-to-use IFNγ ELISA. Stimulation was carried out by adding 250 µl blood per stimulation tube. After 21-23 hours incubation at 37°C, plasma supernatants were collected and IFNγ concentrations assessed by ELISA. All plasma samples were assayed in 4-fold dilution. Concentrations were calculated using a standard curve obtained by four parameter logistic curve fitting. Negative control responses that were too low to be calculated were assigned a concentration of 0.6 pg/mL, which is the limit of quantification of the ELISA. Concentrations above the range of the standard curve were assigned 105% of the concentration of the highest standard (500 pg/mL) multiplied by the dilution factor, resulting in a cut-off at 2100 pg/mL. A subject was scored positive by the new IGRA format if negative and positive controls met the quality cut-offs (**Supporting Information Part 2** and **Supporting Figure S7**), the *C. burnetii*-induced IFNγ production was ≥10 pg/ml above background and the stimulation index (SI) of the *C. burnetii*-specific response was ≥10 (**Supporting Information Part 2** and **Supporting Figure S8**).

### Statistical Analysis

Statistical analysis was performed using GraphPad Prism v8 (San Diego, CA, US) and using the Graphpad McNemar calculator (https://www.graphpad.com/quickcalcs/mcNemar2/).

## Results

### Cellular Responses to Heat-Killed *C. burnetii* Are Independent of the Strain Used for Whole Blood Stimulation

**Figure 1:**
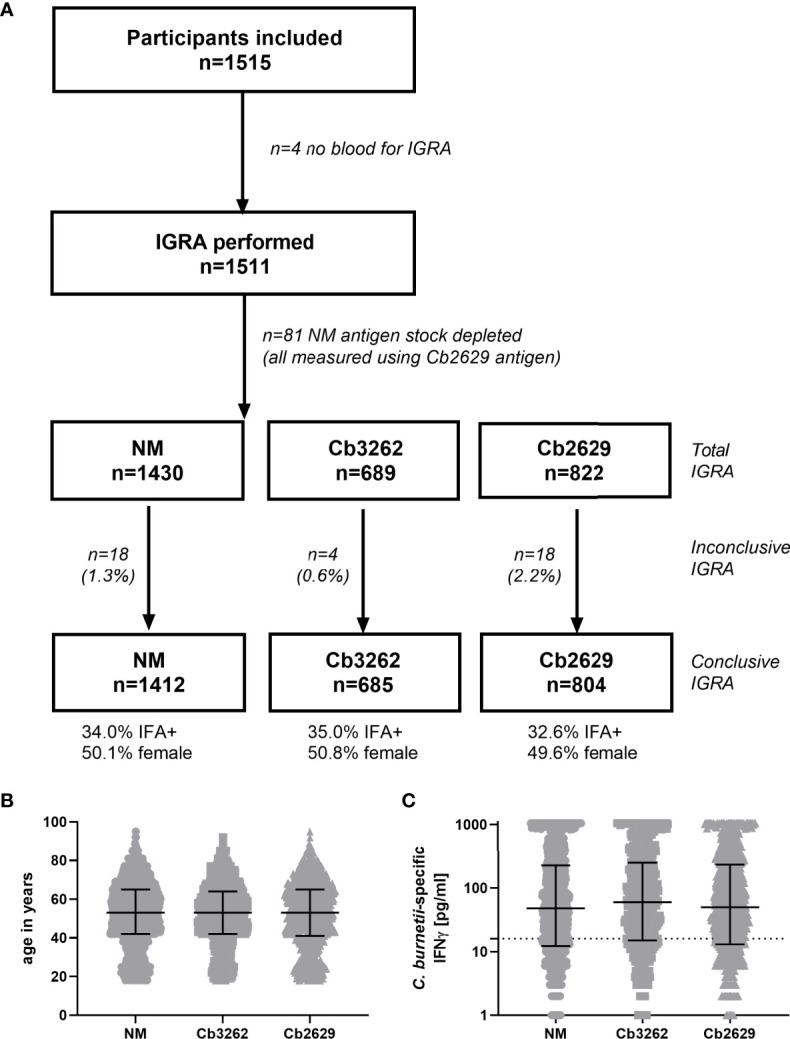
Flowchart, demographics and magnitude of *C. burnetii-*specific IFNγ responses of subjects analyzed by IGRA using the three different strains of *C. burnetii*. **(A)** Flowchart of subjects subjected to IGRA stimulations with the three *C. burnetii* strains including proportion of gender and IFA positivity amongst subjects with a conclusive IGRA result. **(B)** Age distribution and **(C)** *C. burnetii*-specific IFNγ response (background-corrected) in individuals with a conclusive IGRA result for NM (n=1412), Cb3262 (n=685) and Cb2629 (n=804). B and C show individual data with medians and interquartile ranges. The dashed line indicates the positivity cut-off of 16 pg/mL for *C. burnetii* specific IFNγ production.

IFNγ responses to *C. burnetii* were assessed in a large cross-sectional population study termed ‘Q Herpen II’, which involved 70% (1511/2161 individuals) of the adult population of the village of Herpen (24, 25). This village experienced a high incidence of *C. burnetii* infection during the 2007-2010 Q fever outbreak (26), with a reported sero-prevalence of 33.8% (25). IFNγ responses were assessed using three different heat-killed antigen preparations, the reference strain *C. burnetii* Nine Mile (NM) RSA 493 (22) as well as two *C. burnetii* strains isolated from the placenta of a goat infected during the Dutch outbreak, Cb2629 and Cb3262. The majority of blood samples (1430/1511) was tested in whole blood stimulations with two strains (NM and Cb2629, n=741; NM and Cb3262; n=689), and 81 blood samples were stimulated with Cb2629 only, due to limited reagent availability (**Figure 1A**). For a number of donors, the IGRA result was deemed inconclusive, due to either elevated background IFNγ production, an insufficient positive control response or insufficient sample volume to run the positive control (**Figure 1A** and **Supporting S5**). Of note, amongst these inconclusive samples, those that did meet the cut-off for a positive *C. burnetii*-specific IFNγ response were largely positive by IFA (7/8), while those that did not meet the cut-off were largely also negative by IFA (9/12) (**Supporting Figure S5**). For all further analysis in this cross-sectional cohort from Herpen, comparisons for cellular IFNγ and serological responses were only made amongst those donors with conclusive IGRA results (n=1412 for NM, n=805 for Cb2629 and n=685 for Cb3262). These three groups had comparable proportions of IFA+ individuals (34.0, 32.6 and 35.0% for donors tested for NM, Cb2629 and Cb3262, respectively) and were highly similar in age (p=0.99 for NM *vs.* Cb2629 and Cb3262 groups, p=0.65 for Cb2629 *vs.* Cb3262 groups; Kolmogorov-Smirnov test) and gender composition (**Figure 1B**).

IFNγ responses to *C. burnetii* were highly similar independent of whether the reference Nine Mile strain or the related Dutch isolates Cb2629 or Cb3262 were used for stimulation (**Figure 1C**). The NM batch was produced in 2010 and grown on BGM cells. In contrast, Cb2629 and Cb3262 were cultured in 2014 under cell-free conditions. Different growth media were used for preparation of the antigens and both Dutch isolates were free from possible BGM cell debris. There was no significant difference in the distribution of IFNγ responses for the three *C. burnetii* strains (p=0.82 NM *vs.* Cb2629; p=0.28 NM *vs.* Cb3262; p=0.66 Cb2629 *vs.* Cb3262; Kolmogorov-Smirnov test), and for individuals tested with two different strains in parallel, responses correlated strongly (Spearman p<0.0001; r=0.95 for both Cb2629 and Cb3262 compared to NM; **Supporting Figure S6**). This indicates that IFNγ responses are driven by specific *C. burnetii* antigens that are present in comparable composition and/or quantity in all tested strains of *C. burnetii* and independently of how these strains were cultured.

### Cellular IFNγ Responses to *C. burnetii* Are More Prevalent Than Antibody Responses in a Highly Exposed Cohort

**Figure 2:**
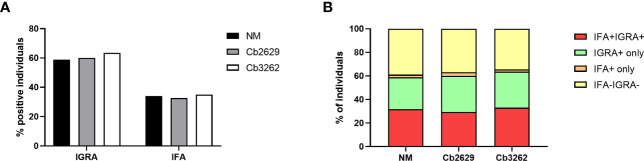
Proportion of donors tested positive by IFA and IGRA. The proportion of samples testing positive by IFA and/or IGRA is shown per group for donors with conclusive IGRA results (n=1420 for NM, n=805 for Cb2629 and n=685 for Cb3262). **(A)** Total proportion of IGRA+ and IFA+ donors per group. **(B)** Proportion of donors with responses by IGRA and IFA; IGRA only; IFA only or none.

**Figure 3:**
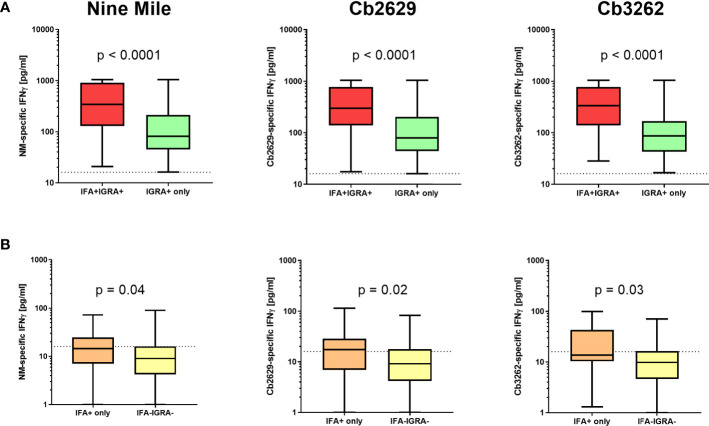
*C. burnetii*-specific IFNγ response induced by Nine Mile, Cb2629 and Cb3262. *C. burnetii*-specific responses (background corrected for negative controls per donor) are shown for individuals that scored positive **(A)** or negative **(B)** by IGRA depending on whether individuals were sero-positive by IFA (red and orange) or not (green and yellow). Groups were compared by Mann-Whitney rank test. The dashed line indicates the positivity cut-off of 16 pg/mL for *C. burnetii* specific IFNγ production. Whisker box plots show boxes with medians and interquartile ranges and whiskers extending from min to max values.

Independent of the *C. burnetii* strain used for stimulations, the prevalence of donors with a positive IFNγ response was considerably higher than the prevalence of seropositive responses by IFA (58.9, 60.0 and 63.5% for donors tested for NM, Cb2629 and Cb3262, respectively; **Figure 2A**). The IGRA identified the vast majority of IFA+ donors (93.3, 90.1 and 94.2% for donors tested for NM, Cb2629 and Cb3262, respectively), while IFA failed to identify half of the donors with a detectable IFNγ response (46.1, 48.0 and 51.0% for donors tested for NM, Cb2629 and Cb3262, respectively) (**Figure 2B**). When accounting for all individuals positive by IGRA or IFA or both, IGRA therefore identified a significantly larger proportion than IFA (95-97% compared to 52-56%; McNemar’s test p < 0.0001; odds ratio (95% confidence interval) 12.0 (8.3-17.8), 9.5 (6.3-14.8) and 14.9 (8.7-27.8) for NM, Cb2629 and Cb3262, respectively). There was a considerable overlap in the magnitude of *C. burnetii*-specific IFNγ responses between individuals that were positive by IGRA only and those that were positive by both IFA and IGRA (**Figure 3A**). Nevertheless, *C. burnetii*-specific IFNγ responses were overall stronger in donors that had also a detectable antibody response by IFA, which was true irrespective of the *C. burnetii* strain used and even found for those individuals that did not meet the positivity cut-off by IGRA (**Figure 3B**). Finally, n=178 participants with a conclusive IGRA result reported symptoms consistent with Q fever in the time frame up to 2014. Out of these, 149/178 (83.7%) had a positive IGRA result based on either Cb2629 or Cb3262, and 135/169 (79.9%) tested using NM. In contrast, only 113/178 of these past symptomatic individuals (63.5%) were positive by IFA.

### Cellular IFNγ Responses to *C. burnetii* Are More Durable Than Antibody Responses in Known Exposed Individuals

**Figure 4:**
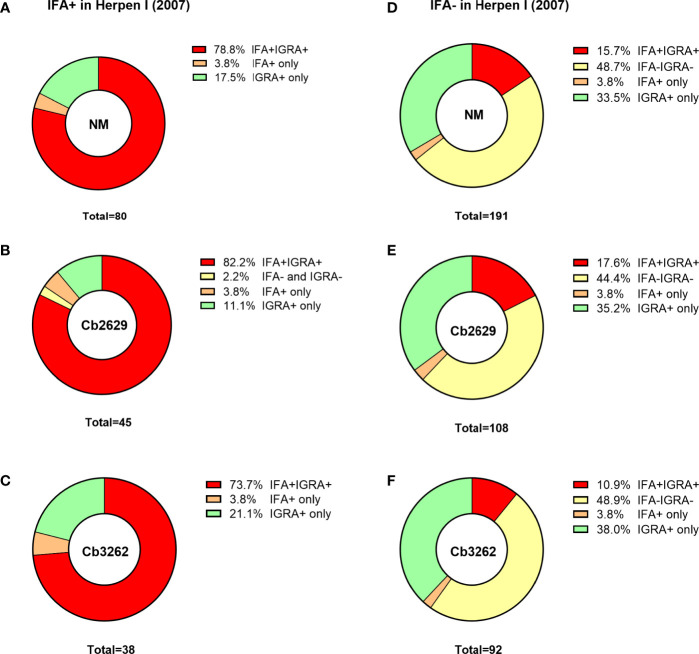
IGRA and IFA results in individuals with known prior Q fever serology status in 2007. The proportion of individuals that scored positive or negative by IFA and IGRA in 2014 [shown separately for Nine Mile **(A, D)**, Cb2629 **(B, E)** and Cb3262 **(C, F)**] was analyzed for individuals with known prior IFA status (IFA+ A-C; IFA- D-F) in 2007.

**Figure 5:**
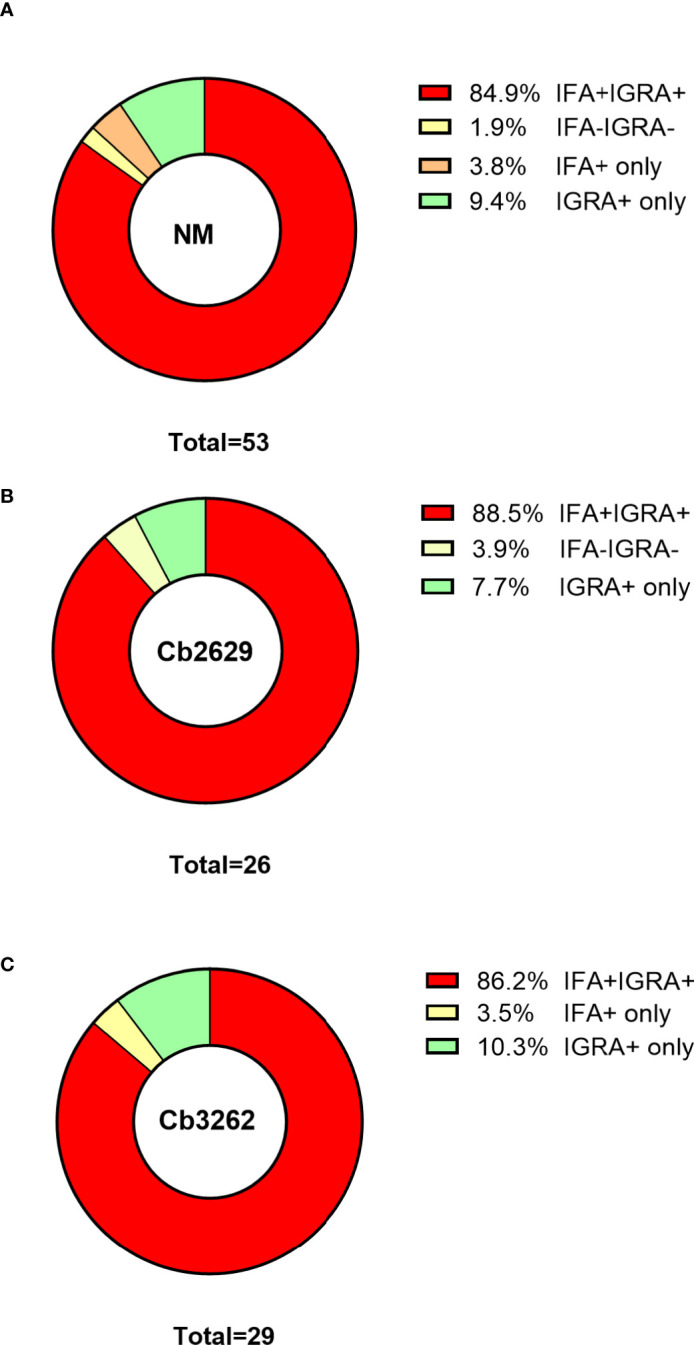
IGRA and IFA results in individuals with notified Q fever. The proportion of individuals that scored positive or negative by IFA and IGRA results [shown separately for **(A)** Nine Mile, **(B)** Cb2629 and **(C)** Cb3262] was analyzed for individuals who were reported to the Municipal Health Service with Q fever between 2007 and 2010.

One possible explanation for the higher detection rates by IGRA compared to IFA is that (detectable) antibody responses might be more short-lived than cellular IFNγ memory responses. To test this hypothesis, data about prior exposure to *C. burnetii* are required. The cross-sectional cohort included n=287 participants that had previously been screened by IFA in 2007 (26): amongst this group were n=271 of individuals with a conclusive IGRA result upon stimulation with NM, as well as n=153 and n=130 with a conclusive IGRA result from Cb2629 and Cb3262 stimulations, respectively (**Figure 4**). In the subgroups assessed by IGRA with NM, Cb2629 and Cb3262, IGRA identified 94-96% of all individuals that were IFA+ in 2007, while only 79-87% remained IFA+ (**Figures 4A–C**). For NM stimulations, the group of individuals that was IFA+ in 2007 was sufficiently large for statistical analysis (n=80), which showed that IGRA was significantly more sensitive than IFA to identify past exposure in previously IFA positive individuals (McNemar’s test p = 0.015; χ^2^ = 5.9; odds ratio 4.7). Amongst the n=191 individuals that remained IFA- in 2007, n=98 (51%) of those screened in 2014 by NM IGRA exhibited either an IGRA response or a positive IFA titer or both. Within this group, the proportion of individuals converting to exposure-positive by IGRA was a lot larger than those converting to positive by IFA, with 96% compared to 35%, respectively (**Figures 4D–F**). The cross-sectional cohort further included n=57 individuals that were reported to the GGD as notified Q fever cases between 2007 and 2010. Amongst this group were n=53, n=29 and n=26 individuals with a conclusive IGRA result upon stimulation with NM, Cb2629 and Cb3262, respectively. In these subgroups, IGRA identified 94-97% of all individuals with notified prior Q fever, while only 88-90% remained IFA+ (**Figure 5**). In both sub-cohorts of individuals with a known prior infection, the IGRA identified the vast majority of individuals that remained IFA+. Therefore, a higher durability of IFNγ responses at least partially accounts for the greater prevalence of positive IGRA responses in this cross-sectional cohort.

### Prevalence and Minimal Specificity Estimate of *C. burnetii*-Specific IGRA Responses in a Low Q Fever Incidence Area

**Figure 6:**
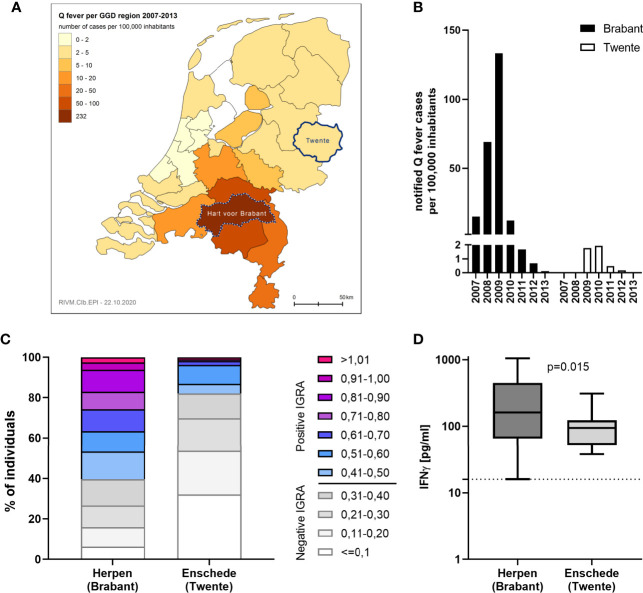
IGRA results in areas with past high and low Q fever prevalence. *C. burnetii*-specific responses were assessed by IGRA *via* stimulation with Cb2629 in n=804 individuals in the high prevalence area of Herpen and the low prevalence area of Enschede in 2014. **(A)** Map showing the cumulative incidence of notified Q fever cases per 100,000 inhabitants from 2007-2013 in the different Municipal Health Service (GGD) regions. The GGD regions where Herpen (Hart voor Brabant) and Enschede (Twente) are located, are highlighted. **(B)** Yearly incidence of notified Q fever cases per 100,000 inhabitants in the years 2007-2013 in the GGD regions Hart voor Brabant and Twente. **(C)** CoxRRs for all individuals are shown. **(D)** Background corrected *C. burnetii*-specific IFNγ responses are shown for n= 482 individuals in Herpen and n=19 in Enschede, who scored positive by IGRA. Responses were compared by Mann-Whitney rank test. The dashed line indicates the positivity cut-off of 16 pg/mL for *C. burnetii* specific IFNγ production.

IGRA responses were additionally assessed using strain Cb2629 in a separate study in Enschede, a city with a much lower Q fever incidence during the 2007-2010 outbreak than Noord-Brabant. Enschede is located in the Dutch Province of Overijssel and the GGD region of Twente, which registered only 27 officially notified cases of Q fever from 2007-2013 (4,33 per 100,000 inhabitants), in contrast to 2381 (232,27 per 100,000 inhabitants) the GGD region “Hart voor Brabant”, in which the outbreak village of Herpen is located (**Figure 6A**). In Enschede, most notified Q fever cases were registered in 2009 and 2010 (**Figure 6B**). Out of n=109 individuals in the Enschede cohort, 106 had a conclusive IGRA result. In line with the expected lower exposure levels in this area, only 18% (19/106) had a positive IGRA response to Cb2629, compared to 60% in Herpen (482/804; **Figure 6C**). Those with a positive IGRA response produced significantly lower levels of *C. burnetii*-specific IFNγ in this low incidence area (p=0.015, **Figure 6D**), and the proportion of individuals with a very high IGRA response (CoxRR > 0.8) was a lot lower in the low incidence area (0.9% in Enschede *versus* 17% in Herpen). Amongst the IGRA positive individuals in Enschede, n=3 (16%) were seropositive by IFA, and another n=9 were seropositive using a more sensitive Cb2629 immunoblot (47%). When assuming that the immunoblot has a sensitivity of 90% (i.e. missing 1 additional donor) and that the n=10 remaining IGRA positive donors were indeed false positives (9.4%), the estimated specificity of the IGRA based on the results in this low incidence area would be at least 90%.

### A New Ready-to-Use IGRA Format to Detect *C. burnetii*-Specific IFNγ Responses Is Highly Sensitive to Detect Past Exposure After 10-14 Years

One hurdle to implementation of the standardized *C. burnetii* IGRA used in the Q Herpen II study in a routine diagnostic setting, e.g for pre-vaccination exposure screening, is its relatively high labor- and time-intensiveness. Therefore, a new ready-to-use format of the IGRA was developed to simplify both the stimulation and ELISA steps. The sensitivity of this new ready-to-use IGRA format was assessed in early 2021 in a cohort of n=95 individuals with known prior exposure status, n=84 of which had previously participated in the 2014 Q Herpen II study. Within this 2021 cohort, n=13 individuals had previously tested negative by both IFA and IGRA and all remained IGRA negative six years later. Another n=4 individuals had tested positive by IGRA in 2014, but did not meet positivity criteria when re-tested in 2015; 3/4 were also negative by IGRA in 2021, while one individual showed a very low positive response (Cb2629-specific production 12 pg/mL). Of the remaining n=78 individuals with known prior exposure, n=45 had previously tested positive by both IGRA and IFA, n=22 had tested positive by IGRA but not IFA in 2014, and n=11 individuals had been initially tested by IGRA or IFA only.

**Figure 7:**
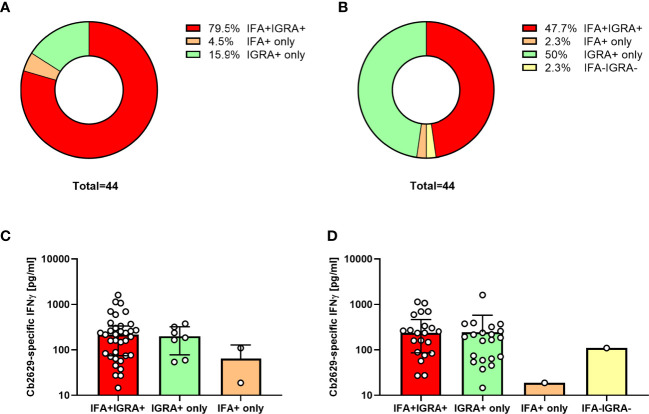
IGRA and IFA results 10-14 years after initial *C. burnetii* exposure. *C. burnetii*-specific responses assessed in using the ready-to-use IGRA format (strain Cb2629) and IFA were compared for n=44 individuals with known prior exposure based on both cellular and humoral immunity (positive IGRA and IFA in 2014/2015), and a conclusive IGRA response in 2021. Donors were scored as IFA positive using an IgG phase II cut-off titer of 1:64 **(A, C)** or the official IFA criteria (IgG phase II titers ≥1:256 in case of no detectable phase I IgG; **B, D**). Data are shown as the proportion of individuals that scored positive or negative by IFA and IGRA **(A, B)** or as the median *C. burnetii*-specific IFNγ response and error bars indicating the interquartile range **(C, D)**. Note that one of the donors treated as IGRA negative in this analysis had a borderline IGRA result (SI 5.0, Cb-specific IFNγ response 109 pg/mL; IFA titer 1:128).

Amongst these *C. burnetii* pre-exposed individuals, 75/78 had a conclusive result and 69/75 met the positivity criteria of the new IGRA format, equating to a sensitivity of 92% to still detect cellular IFNγ responses 10-14 years after initial exposure. One additional donor was borderline by IGRA with a SI = 5.0 and a *C. burnetii*-specific IFNγ production of 109 pg/mL. Within the subgroup of n=44 individuals that were positive by both IGRA and IFA in 2014 and had a conclusive IGRA result in 2021, this proportion was even higher, with 95.5% (42/44) remaining IGRA positive and another n=2 borderline by IGRA (**Figure 7A**). In contrast, only 84% (37/44) remained positive by IFA when using the same cut-off as used in Q Herpen II (IgG phase I or II titer of ≥1:64; **Figure 7A**). When using the official FocusDX interpretation criteria of only scoring an IFA positive for IgG phase II titers ≥1:256 in case of no detectable phase I IgG, only 50% (22/44) of these individuals met cut-off for a positive IFA result (**Figure 7B**). Of note, there was no difference in the magnitude of *C. burnetii*-specific IFNγ responses between those individuals that did or did not remain positive by IFA regardless of the IFA cut-off used (**Figures 7C, D**).

## Discussion

Using a standardized *C. burnetii* IGRA, we assessed its performance in comparison to serological detection in an area of high Q fever incidence up to seven years after initial exposure during the Dutch Q fever outbreak 2007-2010. In a cohort of >1500 individuals from the Dutch outbreak village of Herpen, we show that 60% of the village population had mounted and retained IFNγ responses to *C. burnetii*. This proportion was independent of the *Coxiella* strain used for stimulation and much higher than the proportion of sero-positive individuals. Moreover, *C. burnetii*-specific IFNγ responses were found to be more durable than antibody responses in two sub-groups of individuals known to have sero-converted by 2007 or previously reported to the municipality as notified Q fever cases. A simplified ready-to-use version of the IGRA identified a larger proportion of pre-exposed individuals as positive than did IFA when assessed 10-14 years after initial exposure. Altogether, these data indicate that IFNγ responses are a more sensitive and durable marker of exposure to Q fever than are serological responses.

Sensitivity and specificity are two important hallmarks of any diagnostic assay. The difficulty of assessing these measures in the context of endemic infectious diseases is to have a known true positive (exposed) and negative (unexposed) population. In addition, in the context of immune responses the additional variable of time comes into play with immune responses, particularly those measurable in the general circulation (antibodies and effector T-cells), contracting over time. For serological detection of *Coxiella* exposure, IFA is considered the gold standard. However, previous research has shown repeatedly that not all individuals who are exposed by vaccination or infection sero-convert (9, 23), that in those who do mount an antibody response these levels decline over time (9, 16, 17, 23, 33, 34) and consequently that nearly 20% of initially seropositive individuals sero-revert in a time frame of 4-7 years (16, 17, 25). Given that cellular and humoral responses are two communicating but nevertheless separate arms of the adaptive immune system, simply using seropositivity as a benchmark for determining assay sensitivity is insufficient. Nevertheless, when using individuals that were seropositive 4-7 years after the Dutch outbreak as a reference, the IGRA has a sensitivity of 90-94%. For individuals with a known serological response in 2007 or official notification as a Q fever case, the sensitivity was even higher at 94-97%.

However, IGRA also identified nearly twice the number of individuals as previously exposed to *C. burnetii* than were identified by sero-conversion based on IFA. This was even more striking in a subgroup of individuals that were still seronegative in 2007, with nearly three times as many converting to exposure-positive by IGRA as compared to IFA. In contrast, only a small minority of donors was identified by IFA only. Furthermore, IGRA also identified a much larger proportion of individuals as previously exposed amongst those subjects that recalled a disease episode consistent with Q fever, but tested negative by IFA. Higher detection rates by IGRA compared to IFA are consistent with historical studies which have shown that following vaccination with the Australian inactivated whole cell vaccine Q-VAX, proliferative cellular responses were more long-lived than serological responses (9, 35): Despite an initial sero-conversion rate of ~80%, only 55-65% of individuals remained seropositive by at least one of multiple measures (competition radioimmunoassay, complement fixation test or immunofluorescence) (9), while >90% individuals still had cellular responses up to 5 years post vaccination (35). In a small cohort study of 16 US CDC employees that had received formalin inactivated Q fever vaccines, no such difference between the longevity of antibody and cellular responses assessed by IFA and IGRA was found (36). However, in contrast to the Australian studies which used whole cell formalin-inactivated Henzerling or NM as an antigen for the cellular assay (9, 35), this US study used chloroform-methanol-extracted NM antigens for IGRA stimulations. Other potentially confounding factors were the small size of the cohort, that individuals had received two different vaccines, and that the analysis was not performed longitudinally but cross-sectionally which limits the comparability of serological and cellular IFNγ responses in terms of longevity. Following a Q fever vaccination campaign with Q-VAX in the Netherlands in 2011, the prevalence of cellular responses by IGRA was higher than that of sero-conversion 6 months post vaccination. Moreover, while the majority of responding individuals showed both IFNγ and antibody responses 6-12 months post vaccination, IGRA and IFA also identified populations that only mounted either a cellular or a humoral response (23), as also reported in the US study (36).

One observation in the current study is that IFNγ responses were higher in individuals that had also sero-converted. Such higher IGRA responses in those with a positive IFA result are consistent with prior observations in the Dutch pre-vaccination screening campaign (22). A possible explanation is that in those individuals that mount a strong cellular IFNγ response, most likely reflecting a high level of circulating effector Th1 memory cells detected by IGRA, there may also be a more effective induction of follicular helper T-cells. This specific T-cell subset is fundamental in supporting the generation of humoral immune memory and thus also drives higher levels of circulating antibodies (37).

One clear limitation of our study in this context is that for the majority of individuals assessed, it is simply not known whether they have been indeed exposed to *C. burnetii* in the past, and hence one cannot know whether every single individual with a positive IGRA response but no detectable antibodies by IFA indeed have a truly *C. burnetii*-specific response. However, the cross-sectional cohort included two subgroups of individuals with known exposure based on either the results of a cross-sectional serological survey in 2007 (26) or official report to the GGD based on symptoms and serology or direct detection by PCR in the acute phase. Notably, only a small proportion of the individuals identified here was reported to the GGD, reflecting the fact that the true number of infections outnumbers reported infections at least 10-12-fold due to infections that remain asymptomatic or do not get diagnosed in time and hence fail to meet the national reporting criteria (25, 38). When analyzing these two above subgroups, IGRA identified a greater proportion of individuals as having past exposure than did IFA, and for NM stimulations in previously sero-positive individuals the group size was sufficiently large for this difference to be statistically significant. Therefore, the results in these subgroups mirror the finding in the full cross-sectional cohort. The sero-reversion rate of approximately 20% in individuals identified as sero-positive in 2007 (26) is further fully in line with the sero-reversion rate after 6 years reported for an independent UK cohort (17). That the proportion of individuals that was both IFA and IGRA positive was higher in these sub-groups compared to the entire cohort is not unexpected: Individuals in these specific two subgroups were defined based on the fact that they had already previously tested sero-positive (in either an observational study or by their treating physician), while the larger cohort may also include individuals that initially mounted solely a cellular/T-cell response or only a weak antibody response that already declined below detection levels.

Moreover, sero-negativity by IFA (albeit being the diagnostic gold standard) does not exclude the presence of antibodies. As described in this manuscript, we developed an in-house Western blot which proved to be more sensitive than the IFA. This same Western blot was used in a follow-up study in which we enrolled a subgroup of individuals from the Q Herpen II study and assessed IFNγ responses to only a small but highly specific set of 50 MHC class II-restricted peptides derived from 40 source protein (2.2% of >1800 encoded *C. burnetii* proteins) (39). Unpublished data from this study showed that amongst n=35 IGRA positive individuals that tested negative by IFA, n=22 (67%) tested positive by Western blot. Amongst those individuals that were negative by IFA but positive by Western blot, 2/6 (33%) participants showed responses to 6 or more of 50 assessed peptides, respectively. This proportion was comparable to donors positive by both IGRA and IFA, recognizing 6 or more out of 50 peptides (14/39, 36%). Even more relevant, amongst donors that were negative by both IFA and Western blot, 2/8 (25%) also recognized 6 or more peptides. Therefore, highly *C. burnetii*-specific T-cell IFNγ responses are detectable in a comparable order of magnitude in all three groups and hence irrespective of IFA positivity.

Altogether, these results therefore indicate that a higher durability of cellular IFNγ responses at least partially accounts for the much greater prevalence of positive IGRA responses compared to IFA in this cross-sectional cohort. Additional explanations are that some individuals fail to mount a detectable serological response, and that IFA may not identify all individuals that have indeed sero-converted, as found also here for those individuals in the low incidence area that had *C. burnetii*-specific antibodies detectable by immunoblot but not IFA. Moreover, the IFA cut-off used in this study was ≥ 1:64, while other studies have used a lower cut-off of ≥ 1:32 (16, 34).

Nevertheless, as for any diagnostic test it cannot be excluded that some of the responses detected by IGRA were false positives, for instance due to cross-reactivity of individual responding T-cell clones (but not antibodies) with epitopes from other pathogens. The absence of a true gold standard for prior exposure makes definitive assessment of IGRA specificity even more difficult than assessing IGRA sensitivity (22, 36). Such an assessment would only be possible in a truly unexposed population in an area where Q fever is not endemic and individuals do not travel. Nevertheless, to gain at least an estimate of the minimum specificity of the IGRA, IFNγ responses were also assessed in a second cohort from a low prevalence area. Given the broad dispersion of Q fever over the Netherlands during the outbreak, exposed individuals were also expected in this population. Moreover, since these individuals underwent venepuncture due to health care reasons, this group is not necessarily comparable to a group of completely healthy individuals from a low incidence area. As expected, the proportion of positively scored IGRAs and the magnitude of the responses were a lot lower in this low incidence area compared to the outbreak village of Herpen. Half of those with a positive IGRA response also had detectable antibody responses by IFA or immunoblot. Even when assuming that the remaining ten donors that were positive by IGRA only were indeed false positives, the estimated specificity of the IGRA based on the results in this low incidence area would still be at least 90%. In reality, this specificity of 90% is likely an underestimation, since at least some individuals in this cohort that were positive only by IGRA may have never mounted a detectable antibody response or had sero-reverted over the 4-7 years since initial exposure, as also found for 13-21% of individuals with known positive serology in 2007.

A study conducted during the Dutch pre-vaccination screening campaign showed a correlation between IFNγ responses and skin test results in individuals with borderline serology results, with elevated IGRA responses in those with borderline compared to negative skin test and highest responses in those that were skin test positive (22). Overall, the IGRA identified more individuals as having prior exposure than did the skin test or serology and amongst those individuals who passed the pre-vaccination screen based on IFA and skin test there was a trend for more common local adverse reactions in those with high pre-vaccination IFNγ responses (22). Based on these data, IGRA has been proposed as a more suitable tool for pre-vaccination exposure screening (40, 41). Additional arguments that have been raised for using an IGRA for pre-vaccination screening are that in contrast to IFA, the IGRA has an internal negative and positive control, continuous scale readout and is not prone to inter-operator interpretation differences (22). And in contrast to the skin test, the IGRA does not require a follow-up visit, is not reactogenic and does not boost immune responses (40, 41). Consequently, the Q-detect IGRA was incorporated in a 2016 pilot study to compare detection of prior *C. burnetii* exposure by IGRA, intradermal skin test and four clinically used serological assays (42). Of 25 participants in this exploratory study, one had known prior exposure due to Q fever infection and seven from prior Q-VAX vaccination. Only the IGRA successfully identified all eight of these subjects, supporting the hypothesis that the Q-Detect IGRA offers a more sensitive means of detecting prior exposure to *C. burnetii* than current standard assays (42). Moreover, there was poor correspondence between the other clinically used tests. Follow-up studies in larger groups are now needed to confirm these results and to assess the cut-off with which the IGRA could be implemented for pre-vaccination screening to avoid unnecessarily excluding individuals from vaccination (40). Availability of the new ready-to-use IGRA format will greatly facilitate such studies and real-life implementation of the IGRA in a routine diagnostic setting.

Another aspect in the present study was the comparison of cellular IFNγ responses to different strains of *C. burnetii*. Previous studies have reported that cellular responses to *C. burnetii* can differ depending on the antigen preparation used. In particular the formaldehyde-inactivated preparation of *C. burnetii* Henzerling strain phase I (the Q-VAX vaccine) has previously been shown to induce weaker IFNγ responses than heat-killed NM (22). This could be attributed to a number of factors, such as the actual antigen dose used, the effect of formaldehyde-induced cross-linking/loss of antigen, thiomersal-mediated inhibition of Th1 responses or the strain of *C. burnetii* used for stimulation. Using strains of very different time, country and host origin, we clearly demonstrate that the *C. burnetii* strain origin has no effect on the induced IFNγ response assayed *ex vivo*. The same was true for the culture method and potential cell culture-derived contaminants, since there was no difference in IFNγ responses induced by BGM-cultured NM and cell-free cultivated Cb2629 and Cb3262. Finally, prior research has shown that the IFNγ response to heat-killed antigen preparations such as used here accurately reflect the response to viable *C. burnetii*: heat-killing of *C. burnetii* NM and Cb3262 attenuates only the production of innate cytokines, but has no effect on the release of T cell-derived cytokines such as IFNγ or the down-steam cytokine Monokine Induced by Interferon Gamma (MIG/CXCL9) (43).

Finally, while we did not specifically investigate which fraction of whole cell *C. burnetii* was responsible for IFNγ release, previously published data show a clear response to highly specific peptides from *C. burnetii* proteins in IGRA+ donors (39), with a significantly higher number of recognized peptides in IGRA+ compared to IGRA- individuals, and amongst IGRA+ individuals a significantly higher production of IFNγ in the IGRA in those that recognized 3 or more peptides compared to those that recognized one or only 1-2 peptides. Although a small degree of cross-reactivity of the whole cell *C. burnetii* IGRA with other pathogens cannot be excluded, these data further clearly indicate that IGRA responses occur in individuals which also recognize highly specific *C. burnetii* epitopes. Endotoxins can also contribute to IFNγ release from T-cells and possibly NK cells, indirectly mediated through monocytes and endothelial cells and further exacerbated by other cytokines (44–46). In the context of a whole blood assay, it is thus possible that monocytes sensing *C. burnetii* LPS also contribute to mediating IFNγ release. While we cannot exclude a contribution of NK cells to IFNγ release, preliminary data in our laboratory have shown that CD4 T-cells are the most common and consistent source of IFNγ in this whole blood assay, followed by CD8 T-cells. In any case, the LPS phase state does not affect IFNγ responses, which were shown to be highly similar for formalin inactivated NM antigen expressing either phase 1 LPS or the truncated, culture-induced phase 2 form (47, 48).

In conclusion, in this study we demonstrate that independent of the *C. burnetii* strain used for stimulation, IGRA constitutes a more sensitive means to detect past exposure to *C. burnetii* than the current serological gold standard IFA. In combination with a simplified ready-to-use IGRA version suitable for implementation in routine diagnostic settings, this makes the assessment of cellular IFNγ responses a valuable tool for exposure screening to obtain epidemiological data, and to identify pre-exposed individuals in pre-vaccination screens.

## Data Availability Statement

All relevant data generated for this study are included in the manuscript and the **Supplementary Files**. The raw data supporting the conclusions of this manuscript will be made available by the authors, without undue reservation within the scope allowed by patient privacy regulations, to any qualified researcher.

## Ethics Statement

The studies involving human participants were reviewed and approved by the medical ethics review commitee of the UMC Utrecht and the METC Brabant, NL. The participants provided their written informed consent to participate in this study.

## Author Contributions

Conceptualization and design (2014 Herpen and Enschede cohorts): AG and MK. Conceptualization and design (2021 Herpen and Sanquin cohorts): AS, AG, AES, MP, and MK. Acquired funding and supervised research activities: AG, AES, and MP. Provided critical reagents: HJR. Acquisition of data: MV, AG, AS, and MK. Analysis and interpretation of data: AG, AS, HPD, and MK. Drafting the article: AS. Revision of article: AG, HPD, HJR, and MK. All authors contributed to the article and approved the submitted version.

## Funding

Financial support for the Q-Herpen II study was provided by the Dutch Ministry of Health, Welfare and Sports (VWS) under project number 321632. Statistical analysis by Numerus GmbH in collaboration with Innatoss Laboratories B.V. was funded by the IN2LifeSciences project and by the Interreg IVB North-West Europe program with collaboration incentive ID C30. Development of the ready-to-use IFNγ ELISA was supported by the European Union’s Horizon 2020 research and innovation program under grant agreement number 720480. Assessment of the ready-to-use IGRA version in 2021 in a cohort with known *Coxiella* pre-exposure was supported by contract HDTRA-12010006 from the U.S. Defense Threat Reduction Agency (https://www.dtra.mil) awarded to Massachusetts General Hospital, subcontracted to Innatoss Laboratories B.V. The funders had no role in study design, data collection and analysis, decision to publish or preparation of the manuscript.

## Conflict of Interest

AG is a senior officer and shareholder of Innatoss Laboratories B.V., MK is currently employed and AS and MV were employed by Innatoss Laboratories B.V., which provides diagnostic screening for infectious diseases and has developed and owns the patent for the Q-detect™ IGRA for cellular immunity testing for Q fever. HPD was employed by Numerus GmbH.

The remaining authors declare that the research was conducted in the absence of any commercial or financial relationships that could be construed as a potential conflict of interest.

## Publisher’s Note

All claims expressed in this article are solely those of the authors and do not necessarily represent those of their affiliated organizations, or those of the publisher, the editors and the reviewers. Any product that may be evaluated in this article, or claim that may be made by its manufacturer, is not guaranteed or endorsed by the publisher.
