# Supplementary material for: Whole Blood Interferon γ Release Is a More Sensitive Marker of Prior Exposure to Coxiella burnetii Than Are Antibody Responses
Source: Front Immunol. 2021 Jul 28;12:701811. doi: 10.3389/fimmu.2021.701811 (PMC8356048; doi:10.3389/fimmu.2021.701811)
Supplement: Supplementary file 1 [file Table_1.docx]

**Supporting information**

1. **Determination of Q-detect^TM^ IGRA cut-off parameters**

Initial technical optimization of the IGRA protocol involved evaluation and standardization of stimulation and readout conditions (choice of anti-coagulant, duration of blood storage prior to stimulation, storage after stimulation, choice and validation of a sufficiently sensitive ELISA readout). To allow for high throughput screening in 96 well format, the assay was adapted from its original format and the stimulation volume reduced to 200 µl (compared to at least 0.5 mL used in prior studies (Schoffelen, Herremans, et al. 2013; Schoffelen, Joosten, et al. 2013). Quality cut-offs for positive and negative controls of the IGRA assay and cut-offs for positivity of the *C. burnetii*-specific IFNγ response were determined both based on the limits of quantification of the ELISA read-out and based on IGRA data obtained using a large number of samples (n=1511) collected in 2014 from the population in the village of Herpen, The Netherlands (Morroy et al. 2016).

- 1. **Quality cut-offs for positive and negative controls**

Non-specific IFNγ release was assessed by stimulation with medium only. Background IFNγ levels were below the dilution factor-corrected limit of quantification for 94.6% of individuals (1429/1511 subjects <16 pg/mL), and IFNγ levels were <40 pg/mL in 98.7% of individuals (1492/1511 subjects) (**Supporting Fig S1**). Since higher levels might indicate excessive baseline activation which may obscure or confound *C. burnetii*-specific responses, individuals with negative control IFNγ levels > 40 pg/mL are reported as inconclusive. For all other donors, *C. burnetii*-specific IFNγ production was calculated by subtraction of non-specific IFNγ release.

Phytohemagglutinine (PHA) was selected as a positive control based on the original publication. (Schoffelen, Joosten, et al. 2013); however, the M-form of PHA (Thermo Fisher, Cat. No. 10576015) was used based on prior experience to be a stronger and more reliably positive mitogen preparation. This positive control was used both to evaluate the validity of the assay as well as a benchmark for the *C. burnetii*-specific response: The quality cut-off of the positive control was set based on the limit of quantification of the ELISA (7.8 pg/mL) used to quantify the response, resulting in a rounded cut-off of 40 pg/mL at a 5-fold dilution of the PHA-stimulated sample. Only 9/1511 individuals tested (0.6%) had a PHA response of < 40 pg/mL (**Supporting Fig S1**). In addition, the PHA-response was required to be at least 10-fold higher than the non-specific background response. Only 10/1511 donors did not meet this criterion, n=7 of which had a too low PHA-response and 2 of which had a too high background response.


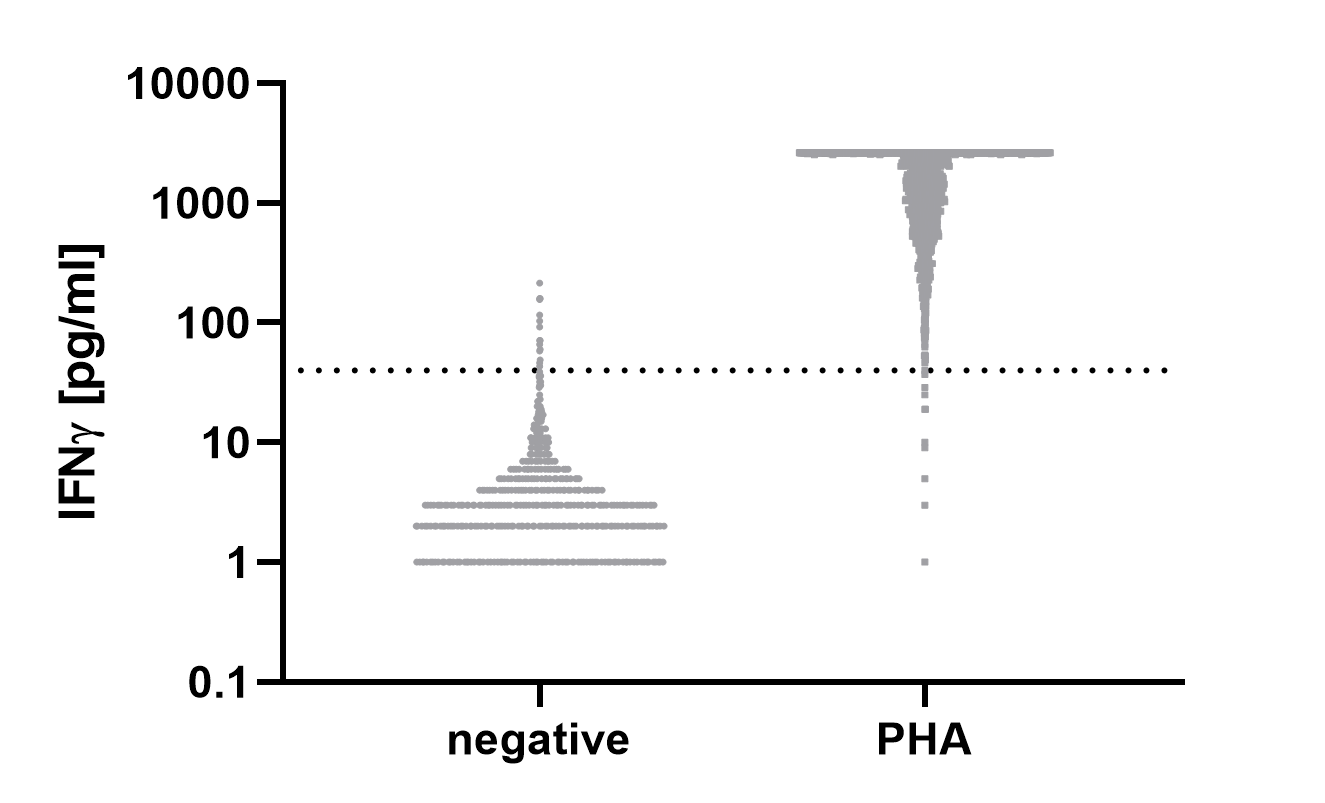


**Figure S1: Background and PHA-induced IFNγ production.** IFNγ responses in negative control stimulations and to the positive control stimulus PHA are shown for n=1511 donors. The dashed line indicates the quality cut-off of 40 pg/mL for both controls. The upper detection limit of IGRA was 2625 pg/mL for the positive control.

- 1. **Interpretation of *C. burnetii*-specific responses**

The minimum cut-off for a *C. burnetii*-specific IFNγ response to be considered positive was set based on the limit of quantification of the ELISA (7.8 pg/mL), resulting in a rounded cut-off of 16 pg/mL at a 2-fold dilution of the *C. burnetii*-stimulated sample. *C. burnetii*-specific IFNγ responses were calculated by subtracting the negative control background response from the IFNγ response measured in the *C. burnetii*-stimulated sample. To avoid falsely scoring a sample positive that meets all the criteria above, but has only a low specific response in combination with either a relatively high background or a very strong PHA-response, interpretation of the *C. burnetii*-specific response in relation to both the background and the PHA-induced response was introduced as a second parameter for results interpretation. This was done to at least partially correct for differential responsiveness of donors due to variables such as number of leukocytes per mL of blood, transport conditions and time to stimulation. Log transformation of calculated concentrations was introduced to correct for log-normal distribution of negative control, positive control and Coxiella-specific responses. The relative *C. burnetii*-specific response (CoxRR) was determined using the following equation: CoxRR = (log(cox)-log(neg))/(log(pos) – log(neg))*.* A CoxRR of ≥ 0.4 was deemed positive. For CoxRRs from this value onwards, the proportion of IFA+ individuals increased considerably (**Supporting Fig S2**). The CoxRR directly correlated with the magnitude of the *C. burnetii*-specific IFNγ production (Pearson p < 0.0001; R^2^=0.54; **Supporting Fig S3**). However, neither background IFNγ responses nor IFNγ release in response to the positive control PHA differed between subjects with negative/low, intermediate or high *C. burnetii*-specific responses (no significant differences by one-way ANOVA with Tukey’s multiple comparison post-hoc test; **Supporting Fig S4**).


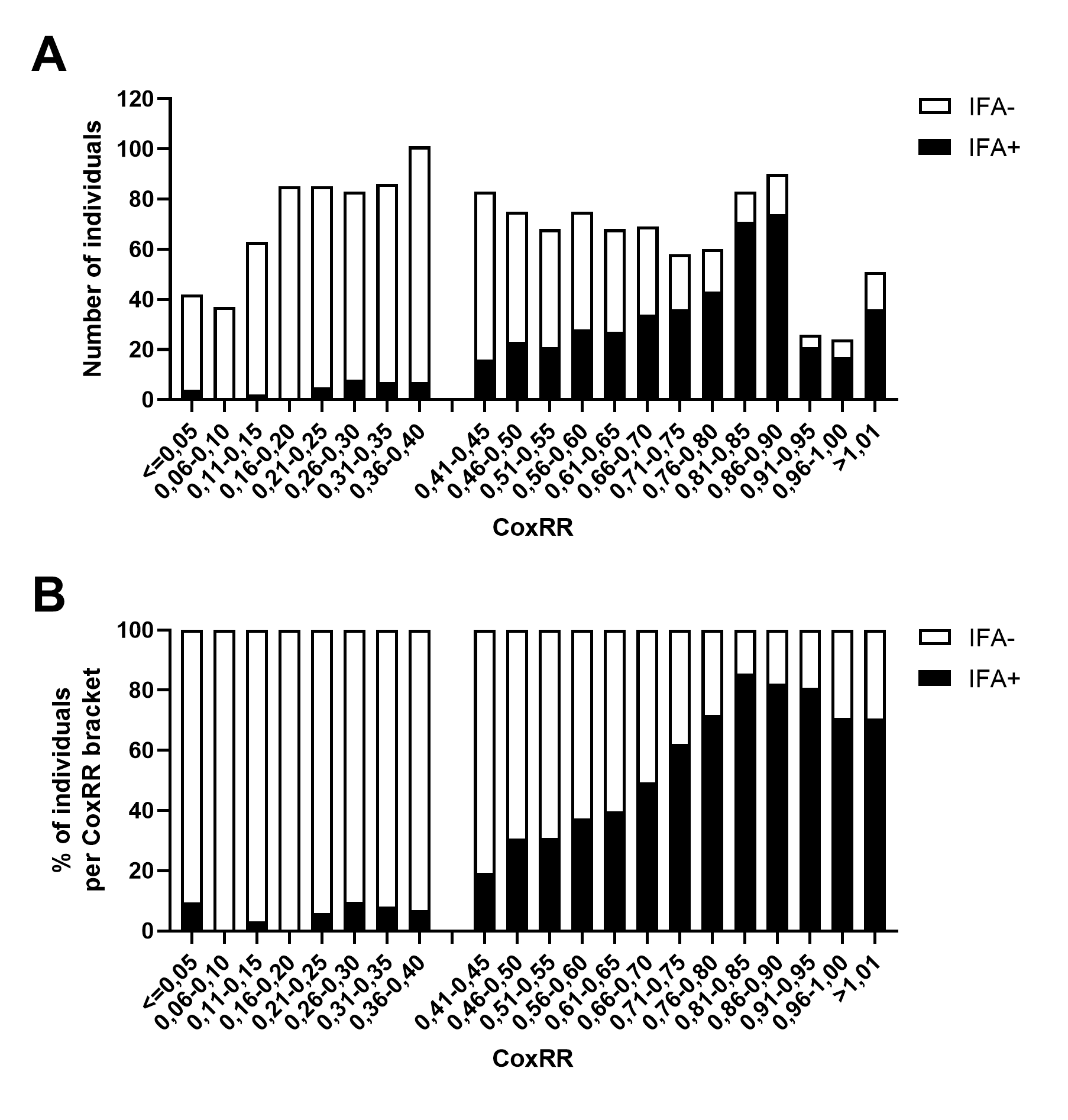


**Figure S2: Proportion of IFA+ and IFA- individuals in the brackets of relative** ***C. burnetii*-specific responses (CoxRR)**

**Figure S3: Association between *Coxiella*-specific IFNγ production and the relative** ***C. burnetii*-specific response (CoxRR) upon stimulation with Cb2629.** Background-corrected IFNγ production and CoxRR are shown for n=822 individuals assessed by stimulation with Cb2629. The upper detection limit of IGRA was 1050 pg/mL for *C. burnetii*-specific responses.

**Figure S4: Background, PHA-induced and *C. burnetii*-specific IFNγ production in donors with negative/low, intermediate and high relative** ***C. burnetii*-specific responses (CoxRR) upon stimulation with Cb2629.** Donors were categorized based on their CoxRR into low/non-responders (CoxRR <0.4; n=320), intermediate responders (CoxRR 0.4-0.8; n=360) and high responders (CoxRR >0.8; n=139). For n=2 donors, no CoxRR could be calculated due to a lack of PHA-induced IFNγ production.


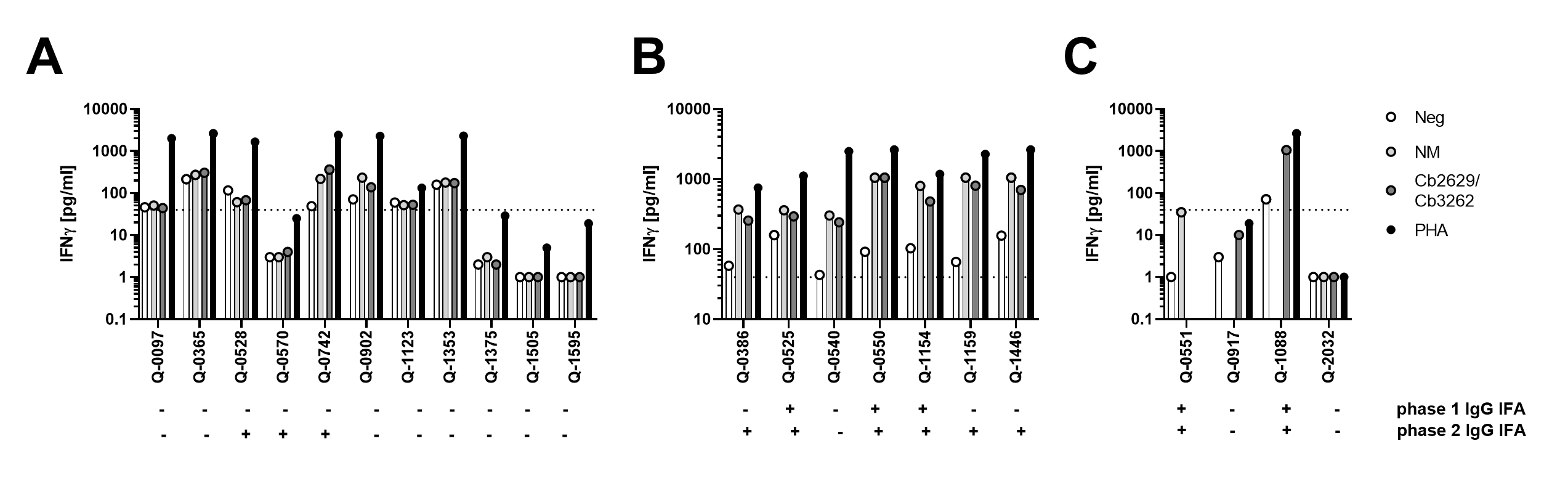


**Figure S5: Donors with inconclusive IGRA results.** IFNγ production in negative control, *C. burnetii* and PHA stimulations is shown for donor with an inconclusive IGRA results due to elevated background or insufficient PHA responses for donors with a Nine Mile (NM) CoxRR (A) ≤0.4, (B) >0.4 and C) those donors for whom no NM CoxRR could be calculated due to missing stimulation conditions. The dashed line indicates the quality cut-off of 40 pg/mL for both negative and positive controls.


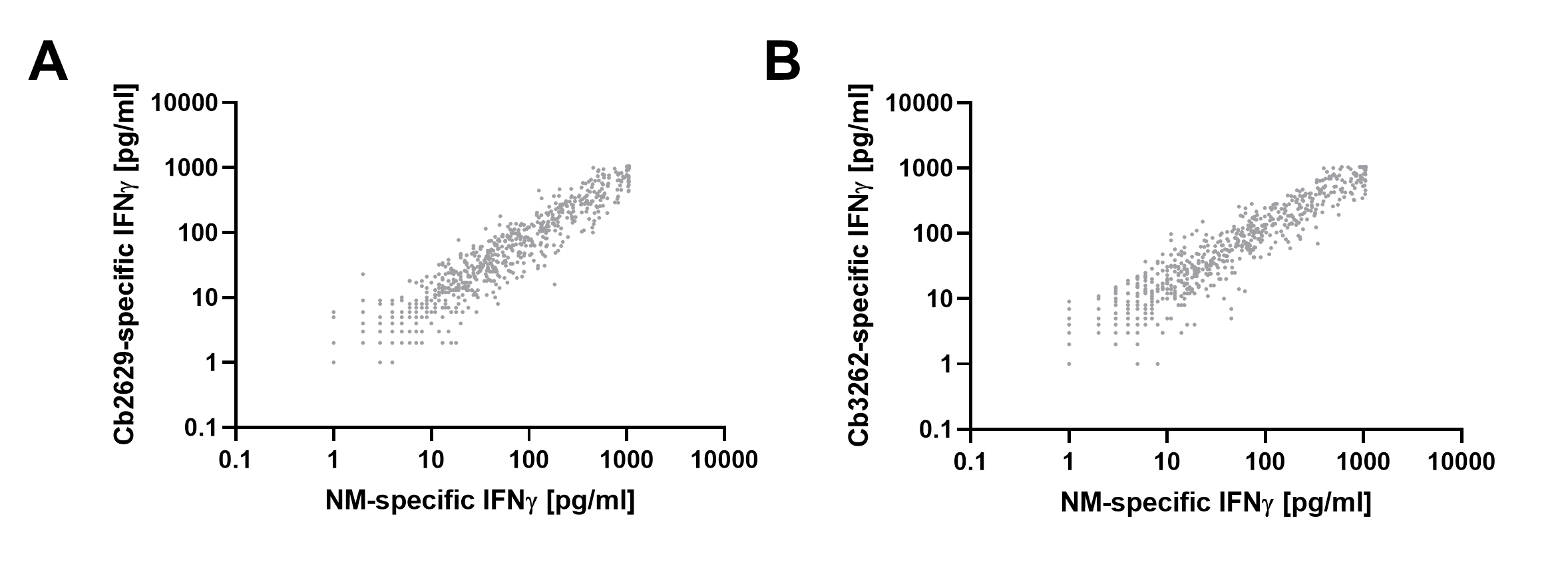


**Figure S6: Association between *Coxiella*-specific IFNγ production in response to NM and Cb2629 or Cb3262.** Coxiella-specific IFNγ production was assessed in parallel upon stimulation with (A) NM and Cb2629 (n=741) or (B) NM and Cb3262 (n=685). Background-corrected responses are shown. The upper detection limit of IGRA was 1050 pg/mL for *C. burnetii*-specific responses.

1. **Determination of ready-to-use IGRA cut-off parameters (Q-detect^TM^ 2.0)**

One hurdle to implementation of the Q-detect^TM^ IGRA in a routine diagnostic setting, e.g for pre-vaccination exposure screening, is its relatively high labor and time-intensiveness. Therefore, a new ready-to-use format of the IGRA (Q-detect^TM^ 2.0) was developed to simplify both the stimulation and ELISA steps. To simplify the stimulation step, microtubes were pre-coated with antigens deposited in in a sucrose matrix, followed by drying. Since PHA was not stable following drying in a sucrose matrix, it was replaced with Staphylococcus enterotoxin B as a positive control. The IFNγ Pelipair ELISA was replaced with a fully validated in-house ready-to-use ELISA, and IGRA samples (negative and positive controls and *C. burnetii* Cb2629-stimulated) were assayed in plasma as opposed to whole blood and all using the same dilution factor. Cut-off criteria for positive and negative controls as well as *C. burnetii*-specific responses were evaluated and determined using blood samples from n=98 de-identified blood bank donors (n=50 males, n=48 females, age range 21-73 years, median age 45 year) who donated blood at the Sanquin blood bank in Amsterdam and Rotterdam in November 2020.

- 1. **Quality cut-offs for positive and negative controls**

Non-specific IFNγ release was assessed by stimulation in tubes coated with sucrose matrix only. Background IFNγ levels were below the dilution factor-corrected limit of quantification for 72.5% of individuals (71/98 subjects <2.4 pg/mL), and IFNγ levels were <40 pg/mL in 95.9% of individuals (94/98 subjects; **Supporting Fig S7A**). Since higher levels might indicate excessive baseline activation which may obscure or confound *C. burnetii*-specific responses, individuals with negative control IFNγ levels > 40 pg/mL are reported as inconclusive. For all other donors, *C. burnetii*-specific IFNγ production was calculated by subtraction of non-specific IFNγ release.

The quality cut-off of the positive control SEB was set to mirror that of the previously determined cut-off for PHA (40 pg/mL), despite the much lower limit of quantification of the new ready-to-use ELISA. Only 1/98 individuals tested (1.0%) had a SEB response of < 40 pg/mL (**Supporting Fig S7B**). In addition, the SEB-response was required to be at least 10-fold higher than the non-specific background response. Only 1/98 donors did not meet this criterion, which was the donor with the too low SEB-response.

- 1. **Interpretation of *C. burnetii*-specific responses**

Prior to validating in blood bank donors the positivity cut-off for a *C. burnetii*-specific IFNγ response (*C. burnetii*-specific response minus background response), this cut-off was pre-defined as the mean + 2x standard deviation of the *C. burnetii*-specific IFNγ response in unexposed donors. Moreover, the upfront decision was made to exclude to top 20% responders in the cohort of blood bank donors, given their unknown history of exposure and the fact that even in a low incidence area such as Enschede (Figure 6), 18% of donors had a detectable T-cell response to *C. burnetii*.

When excluding the top 20% responders, the mean + 2x standard deviation of *C. burnetii*-specific IFN production was 9.05 pg/mL (n=78 donors). The donor with the strongest response in this group had a response of 10.06 pg/mL. Therefore, a rounded cut-off of 10 pg/mL was set for *C. burnetii*-specific IFNγ responses (**Supporting Fig S7C)**.

Notably, the response to the positive control SEB exceeded the upper limit of the ELISA in 19/98 donors and was much higher than the response to a liquid PHA formulation assessed in parallel (**Supporting Fig S7B)**. This greatly impacted the resulting calculated CoxRR values, requiring an adjustment of CoxRR cut-offs to not score an identical *C. burnetii*-specific IFNγ responder negative only due to a new positive control. Therefore, it was decided to treat SEB as a positive control only, similar as QuantiFERON TB (Qiagen), and not to use this response to benchmark the *C. burnetii*-specific readout. Instead, to avoid falsely scoring a sample positive that meets all the criteria above, but has only a low specific response and a relatively high background, interpretation of the stimulation index (SI = *C. burnetii*-specific response/background response) was introduced as a second parameter for results interpretation.

A SI of ≥ 10 was deemed positive and a SI ≥ 3 and <10 was considered borderline.

We re-evaluated the original Herpen-II data using the new criteria. Amongst all IFA+ donors with a conclusive NM IGRA result (n=480), SI ≥ 10 identified slightly less donors (443/480; 92.3%) than a CoxRR ≥ 0.4 (448/480; 93.3%). However, the remaining n=5 donors as well as another n=15 that had a *C. burnetii*-specific IFNγ production of ≥ 16 pg/mL but failed to meet the CoxRR cut-off of 0.4, scored as borderline by IGRA (SI ≥3 and < 10; **Supporting Fig S8A)**. Amongst all subjects deemed NM IGRA positive based on CoxRR ≥ 0.4 in the Q Herpen II cohort, this cut-off of SI ≥ 10 positively identified 799/831 subjects (96.1%); the remaining 32/831 were borderline positive donors (*C. burnetii*-specific IFNγ response 16-214 pg/mL) with a stimulation index between 4.1 and 9.9. On the other hand, SI ≥ 10 identified n=44/153 donors as positive that had a *C. burnetii*-specific response of ≥ 16 pg/mL, but were deemed NM IGRA negative based on a CoxRR < 0.4 (**Supporting Fig S8B)**. The key distinguishing features between those donors identified by SI ≥ 10 only (n=44) or CoxRR ≥ 0.4 only (n=32) was that those identified by SI only had relatively high PHA responses, resulting in a low CoxRR; while those identified by CoxRR only had either a high background IFNγ response (n=9) or a particularly low PHA response, resulting in a high enough CoxRR despite a low *C. burnetii*-specific IFNγ response (**Supporting Fig S8B and C)**. Overall, using SI ≥ 10 resulted in a slightly higher proportion of donors scored positive by IGRA in the Herpen II cohort than when using a CoxRR ≥ 0.4 (SI: 843/1412, 59.7%; CoxRR 831/1412, 58.9%).


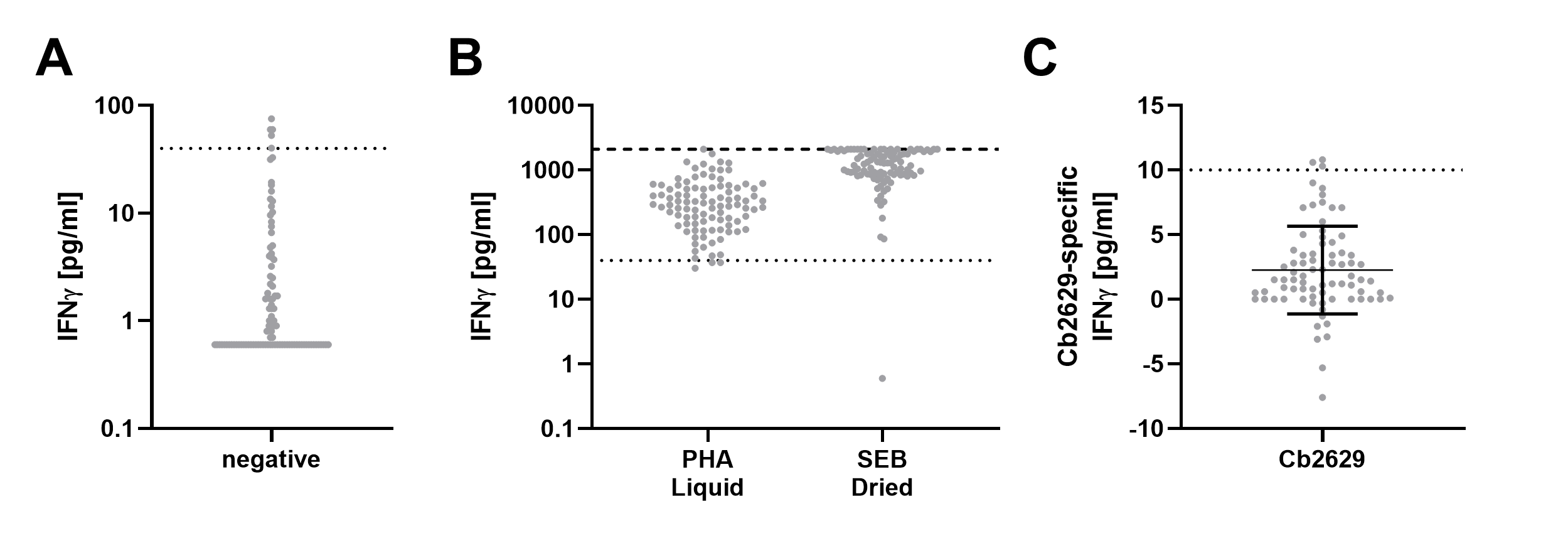


**Figure S7: Background, SEB and Cb2629-induced IFNγ production in blood bank donors.** IFNγ responses in negative control stimulations (A) and to the positive control stimulus SEB (B) are shown for n=98 donors. The thin dashed line indicates the quality cut-off of 40 pg/mL for both controls. The upper detection limit of IGRA was 2100 pg/mL for the positive control (thick dashed line. *C. burnetii* Cb2629-specific IFNγ responses are shown for the bottom 80% responders (n=78) with mean and standard deviation; the thin dashed line indicates the positivity cut-off at 10 pg/mL.

**
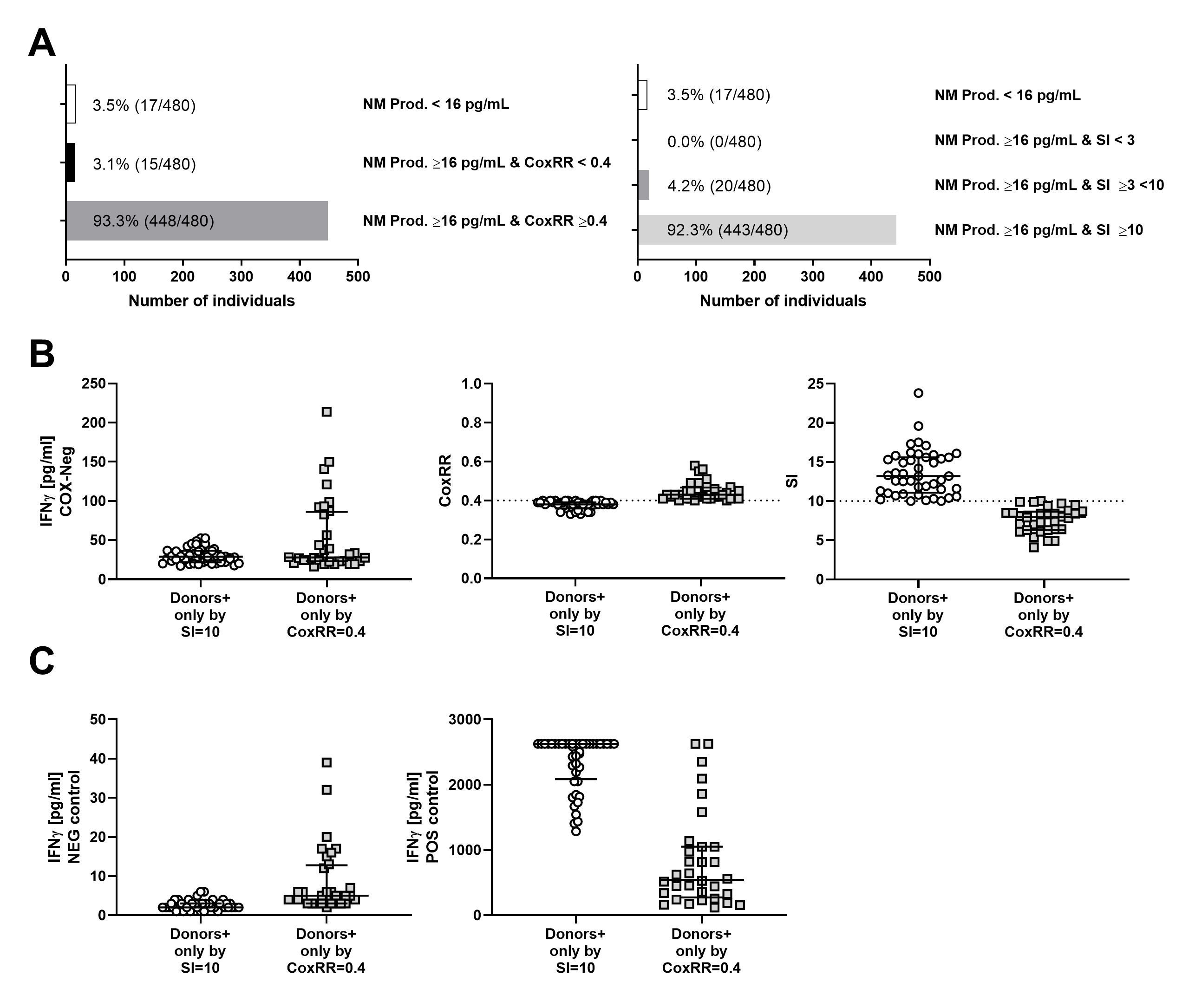
**

**Figure S8: NM IGRA results based on CoxRR ≥ 0.4 or SI ≥ 10 cut-offs in the Q Herpen II cohort.** (A) The number and proportion of individuals meeting the various cut-offs for *C. burnetii*-specific IFNγ responses and either a CoxRR ≥ 0.4 or SI ≥ 10 is shown for n=480 IFA+ subjects with a conclusive NM IGRA result. For those subjects with a *C. burnetii*-specific response of ≥ 16 pg/mL that met a cut-off only by SI ≥ 10 (n=44) or only by CoxRR ≥ 0.4 (n=32), the following parameters were compared: (B) *C. burnetii*-specific IFNγ responses, CoxRR and SI, (C) background and positive control (PHA) IFNγ responses. Data for individual donors are shown with median and interquartile ranges. SI, stimulation index.

**References**

Morroy, G., W. Van Der Hoek, Z. D. Nanver, P. M. Schneeberger, C. P. Bleeker-Rovers, J. Van Der Velden, and R. A. Coutinho. 2016. 'The health status of a village population, 7 years after a major Q fever outbreak', *Epidemiol Infect*, 144: 1153-62.

Schoffelen, T., T. Herremans, T. Sprong, M. Nabuurs-Franssen, P. C. Wever, L. A. Joosten, M. G. Netea, J. W. van der Meer, H. A. Bijlmer, and M. van Deuren. 2013. 'Limited humoral and cellular responses to Q fever vaccination in older adults with risk factors for chronic Q fever', *J Infect*, 67: 565-73.

Schoffelen, T., L. A. Joosten, T. Herremans, A. F. de Haan, A. Ammerdorffer, H. C. Rumke, C. J. Wijkmans, H. I. Roest, M. G. Netea, J. W. van der Meer, T. Sprong, and M. van Deuren. 2013. 'Specific interferon gamma detection for the diagnosis of previous Q fever', *Clin Infect Dis*, 56: 1742-51.
